# Targeted cell ablation-based insights into wound healing and restorative patterning

**DOI:** 10.1016/j.pbi.2019.08.006

**Published:** 2019-12

**Authors:** Lukas Hoermayer, Jiří Friml

**Affiliations:** Institute of Science and Technology Austria, 3400 Klosterneuburg, Austria

## Abstract

Plants as sessile organisms are constantly under attack by herbivores, rough environmental situations, or mechanical pressure. These challenges often lead to the induction of wounds or destruction of already specified and developed tissues. Additionally, wounding makes plants vulnerable to invasion by pathogens, which is why wound signalling often triggers specific defence responses. To stay competitive or, eventually, survive under these circumstances, plants need to regenerate efficiently, which in rigid, tissue migration-incompatible plant tissues requires post-embryonic patterning and organogenesis. Now, several studies used laser-assisted single cell ablation in the *Arabidopsis* root tip as a minimal wounding proxy. Here, we discuss their findings and put them into context of a broader spectrum of wound signalling, pathogen responses and tissue as well as organ regeneration.

## Highlights

• Cell ablation mimics wounding on a single cell level.
• Calcium, ROS, and hormone dynamics were described.
• Local, regional, and systemic responses can be distinguished.

**Current Opinion in Plant Biology** 2019, **52**:124–130

This review comes from a themed issue on **Cell biology**

Edited by **Eva Benkova** and **Yasin Dagdas**

For a complete overview see the Issue and the Editorial

Available online 2nd October 2019

**https://doi.org/10.1016/j.pbi.2019.08.006**

1369-5266/© 2019 The Authors. Published by Elsevier Ltd. This is an open access article under the CC BY license (http://creativecommons.org/licenses/by/4.0/).

## Introduction

Well-studied mechanisms of wound healing in animals rely strongly on targeted migration of cells to the wound area. In plant tissues, this is not possible, since plant cells are encapsulated by their rigid cell walls. Thus, regeneration in plants has to rely on oriented cell divisions, acquisition of new cell fates and on directional cell elongation. Early wounding studies in the 19th and beginning of 20th century provided initial phenomenology of regeneration [1,2,3^•^] but only in the last decade approaches mainly involving the surgical removal of the root tip provided much insight into the mechanism of regeneration and accompanied transcriptional reprograming [4,5^••^]. However, the cellular processes and, in particular, molecular mechanisms underlying this regeneration response remain poorly characterized. Recent studies employing local, targeted cell elimination in the roots of the model plant *Arabidopsis thaliana* promise to provide fresh insights into the still mysterious mechanism of wound healing in plants.

## Non-targeted wounding studies

Most of the earlier wounding experiments involved surgically induced, rather large-scale injuries in different tissues of various plant models. Originally, these studies involved simple observation of processes following the wounding and, later, mainly with the use of *Arabidopsis* root, they employed global transcriptome analysis and more sophisticated use of molecular markers and other genetic tools.

### Cellular responses during regeneration

**Figure 1:**
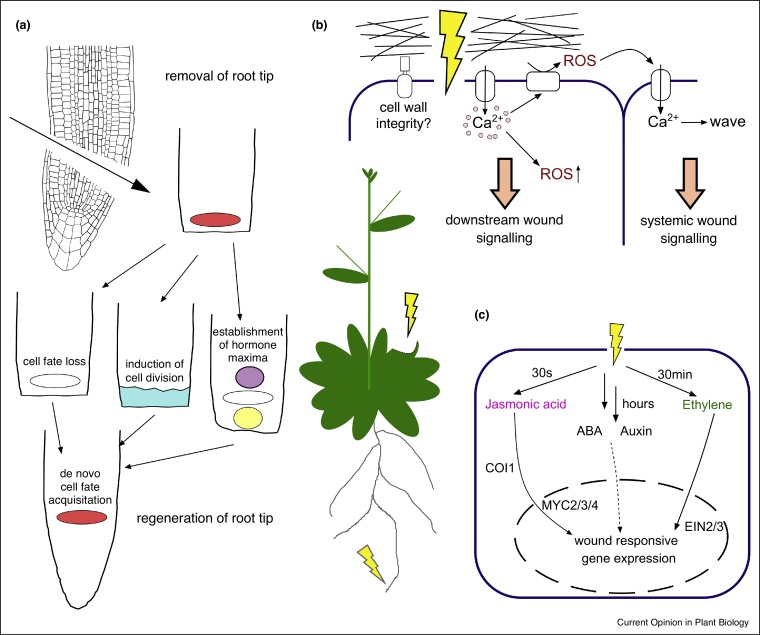
Wounding triggers primary wounding signals, phytohormone signalling and complex regeneration responses. **(a)** Cutting off the root tip including the stem cell niche leads to a complete rebuilding of the missing structures by the following processes: (i) Dedifferentiation in cells close to the wound and adoption of embryonic/stem cell programs [5^••^,^7^]; (ii) Increase in division rates in cells close to the wound and switch in division planes [7]; (iii) Establishment of new accumulation zones for the phytohormones cytokinin (purple) and auxin (yellow) to define the new stem cell niche [5^••^]; (iv) Finally, *de novo* establishment of correct cell types in newly generated cells to restore the original tissue pattern [4,5^••^]. **(b)** Wounding on a cellular level means the disruption of the cellular envelope – cell wall (black) and plasma membrane (blue). Cell wall integrity sensing is presumably involved in wound signalling [20^•^,^21^]. Wound signalling quickly manifests as a Ca^2+^ wave which spreads through neighboring tissues [11,12]. The Ca^2+^ wave relates to the production of ROS in the apoplast and causes itself an oxidative burst inside and outside the cells [13, 14, 15]. Together, Ca^2+^ and ROS trigger multiple downstream signalling events at the wound site and in distal organs to induce immune responses [12,14,20^•^]. **(c)** Wounding induces production of various phytohormones with different dynamics. Jasmonate accumulation starts seconds after the wounding [50] and is perceived by CORONATINE INSENSITIVE1 (COI1) [51]. This leads to the activation of MYC2/3/4 transcription factors regulating downstream genes [52]. Ethylene accumulates 30 min after wounding by an increased activity of its biosynthesis genes [24] and acts through ETHYLENE-INSENSITIVE PROTEIN 2/3 (EIN2/3) transcription factors [53,20^•^]. ABA accumulation after wounding occurs after several hours in desiccated tissues and presumably functions in maintaining healthy plant physiology rather than immune responses [26]. Wounding induces changes in auxin accumulation and signalling after removal of the whole root tip; this involves induction of YUCCA biosynthetic components that play an important role in rebuilding destroyed structures [25,46].

The most obvious response of surrounding cells to wounding is (re)entry into mitosis, also in differentiated cells that have left the cell cycle. These cells dedifferentiate, divide, and form the new cell walls parallel to the wound site ultimately filling the wound with new cells [2,6,3^•^]. In the root meristem, where cells are constantly in the cell cycle, wounding enhances cell divisions in cells close to the wound site; these wound-activated root cells subsequently lose their identity and adopt embryonic/stem cell-like identity (Figure 1a) [5^••^,^7^]. Although these processes have been well described, neither the signal that activates the neighboring cells nor the mechanism coordinating which cells are responsive, has been identified.

Notably, even when the whole stem cell niche of the root is removed, the root meristem pattern is re-established *de novo* with correct arrangement of the lost cell types (Figure 1a) [4]. Single cell sequencing revealed that the newly generated cells quickly adopt the required new cell types, and this is partly dependent on the spatially separated maxima of two major phytohormones, auxin and cytokinin (Figure 1a). However, this *de novo* cell fate acquisition occurred (albeit with less efficiency) also when these maxima were disrupted, which suggests so far unknown intercellular positional signalling that coordinates the re-patterning of the root tip [5^••^]. This highlights the superior ability of plant organs to fully regenerate and restore correct tissue patterns.

### Primary wound signalling

For the efficient initiation of defence responses and regeneration, plants need to quickly recognize the invaders or the induced destruction and signal to the immediate surroundings and the rest of the plant [8^•^,^9^,^10^]. The first known downstream signalling events that occur after herbivore attack or wounding are Ca^2+^ wave initiation [11,12] and an accumulation of reactive oxygen species (ROS) (Figure 1b) [13, 14, 15]. Wounding and pathogen associated elicitors also induce the production of small peptides that act as defence activators [16], for example, Pep1 and Pep2, which activate downstream immune responses against root pathogens [17, 18, 19].

Although these processes are well established to occur after the wounding/herbivore attack and mediate immune responses in plants (for a detailed review see Ref.: [20^•^]), they are triggered by an initial wound signal that is still unknown. Cell wall integrity sensing by constant measurement of the wall composition [20^•^,^21^] is thought to be a crucial element of wound detection. However, no direct, mechanistic connection between the known components of the cell wall integrity sensing and the wound/herbivore responses has been established.

Unsurprisingly, phytohormones, as universal endogenous signals, are induced with different dynamics after attack to contribute to the balance of growth and immunity/defence [22]. Historically, by extracting organic compounds from wound sites, the signalling compound traumatin was isolated which accelerates the wound healing when exogenously applied [3^•^]. Similarly, wounding induces jasmonic acid (JA) [23], ethylene (Et) [24] and less directly, auxin [25] and abscisic acid (ABA) (Figure 1c) [26,27]. While the biosynthetic pathways for most of these phytohormones are known, the exact production sites and the signalling mechanism underlying their activation, have not been investigated.

## Wounding by targeted cell elimination

Recent reports have made use of targeted elimination of a single cell or small group of cells coupled with state-of-the-art live imaging allowing for more precise characterization of the wound responses and regeneration processes.

### Laser ablation technique

In the 90 s, the UV laser ablation technique was introduced allowing for elimination of single cells. Originally, this was used to study cell-to-cell signalling and patterning mechanisms rather than as a tool to induce wounding and study regeneration. This technique has the advantage of removing a cell with spatial and temporal preciseness [28^•^,29^•^], in contrast to genetic [30] or chemical ablations [31^••^,32^••^]. Different types of lasers on different imaging setups [28^•^,29^•^,31^••^,^33^,34^••^] have been used with propidium iodide staining which stains cell walls, allow identification of dead cells and also pre-sensitizes cells for ablation [35]. This allowed the first live observation of wound healing responses in real time and *in situ* [32^••^].

### Cellular responses during regeneration

**Figure 2:**
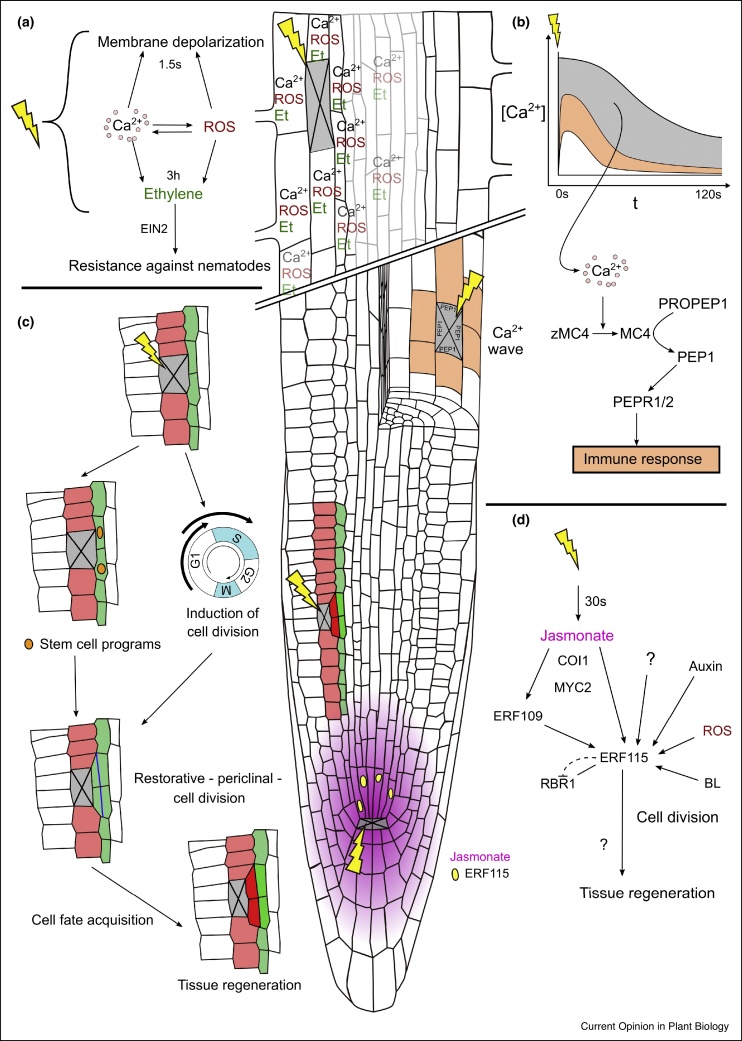
Single cell ablation in the Arabidopsis root meristem triggers multiple local and regional wounding responses. **(a)** Ablation of cortex cells in the elongation zone triggers the induction of Ca^2+^, ROS, ethylene, and membrane depolarization. The increase in Ca^2+^ influx after ablation is dependent on ROS production in the apoplast by RBOH enzymes and allows the fast change in membrane polarization (1.5 s after ablation). Additionally, it induces an accumulation of ROS around the wound that occurs ∼6 min after the ablation. Both, Ca^2+^ influx and ROS production contribute to the ethylene signalling induction by an increased ACC SYNTHASE 6 (ACS6) expression starting three hours after ablation. Eventually, ethylene signalling via EIN2 increases the resistance against nematode infection [37^••^]. **(b)** Laser ablation of epidermis cells in the transition zone triggers a Ca^2+^ influx that spreads throughout the adjacent tissue but results in different amplitudes depending on the distance from the harmed cell. Harmed cells (grey) exhibit a stronger Ca^2+^ influx than those directly adjacent to the eliminated cells (orange) and cells further away (white). Strong influx and complete destruction of membrane integrity activate METACASPASE4 (MC4) from inactive zMC4, which cleaves the PRECURSOR OF PEP1 (PROPEP1) into Pep1. By this, it becomes translocated from the vacuolar membrane to the cytosol to be perceived by the PEPR1 and PEPR2 receptors at the cell surface of neighboring (orange) cells [34^••^]. **(c)** Ablation in the root meristem triggers restorative divisions to replace the eliminated cells. These divisions happen predominately in the inner adjacent cells. They are induced by the activation of stem cell programs (orange nuclei; here: SHR – CYCD6;1) and an accelerated progression through the cell cycle. They include the switch of the division plane from anticlinal to periclinal, and the newly generated outer daughter cells adopt the cell fate of the eliminated cells to eventually regenerate the disrupted tissue pattern [32^••^]. **(d)** Ablations in the stem cell niche trigger a jasmonate induction within 30 s which is perceived by COI1 to activate MYC2, a JA-dependent transcription factor. MYC2 binds to the promoter of ERF115 to enhance its expression around the wound site [41^••^]. ERF115 is also activated by its JA/MYC2-dependent homologue ERF109 [41^••^] and by downstream signalling of auxin [41^••^], ROS [49], and brassinosteroids (BL) [47,48]. In ablations outside the stem cell niche, ERF115 expression is confined to cells directly adjacent to the killed cell [31^••^,32^••^]. ERF115 can bind to RETINOBLASTOMA-RELATED1 (RBR1) and inhibit its activity to regulate the division rate in the quiescent centre and the stem cell niche [41^••^]. Few downstream targets of ERF115 have been identified. One of them, PSK5, might be involved in the acceleration of the cell cycle progression [47]. Eventually, ERF115 transcription factor activity contributes greatly to tissue regeneration after single cell ablation as well as whole root tip removal [31^••^,32^••^,41^••^]. Yellow thunderbolts indicate UV laser ablation.

The root meristem is a tissue where cells are constantly in the cell cycle to proliferate for a sustained growth. Cell elimination dramatically accelerates division rates of adjacent cells predominately at its inner adjacent side, as the time required for one division is reduced from 18 to 5–12 hours (depending on the cell type) (Figure 2c). These ‘restorative divisions’ involve a change in division planes from anticlinal (perpendicular to the growth axis) to periclinal (parallel to the growth axis) allowing for efficient replacement of the dead cells in the wound from the inside. Earlier studies showed that also in the stem cell niche, ablated cells are replaced by irregular divisions of adjacent cells [28^•^]. Outside of the stem cell niche, in differentiating cells, stem cell programs aid the regeneration process as seen by the re-activation of the endodermis/cortex (SHR/SCR and CYCD6;1) or the lateral root cap/epidermis (FEZ and SMB) stem cell regulators (Figure 2c) [32^••^]. Additionally, PLETHORA transcription regulators expressed in a decreasing gradient from the stem cell niche and associated with root stem cell activity [36] appear to endow cells with the competence to induce restorative divisions outside of the stem cell niche [32^••^].

Already the earlier ablation experiments suggested that cells in the root adopt their fate depending on the tissue context [28^•^,29^•^]. This is manifested dramatically during restorative divisions of any cell type. After the division plane switch, the inner daughter cell, which stays in the cell file it originated from, retains its identity. Remarkably, the outer daughter cell rapidly adopts the cell identity of the eliminated cell, which it replaces (Figure 2c) [32^••^].

The restorative divisions, which require accelerated cell cycle progression, division plane switch and finally cell fate change of the daughter cells, appear to be very robust and likely dependent on multiple redundant stem cell program-dependent and independent mechanisms. However, what signal triggers these divisions and what mechanism restricts them to cells only directly adjacent to the wound, remains elusive.

### Primary wound signalling

Similar to herbivore attacks, wounding of single cells in the root meristem induces Ca^2+^ waves in the surrounding tissue. However, harmed cells exhibit a greater Ca^2+^ influx with an increased duration which is translated by a novel Ca^2+^-responsive protease, metacaspase MC4, into the rapid processing and release of Pep1 peptide. Eventually, the secreted Pep1 reaches the surface of neighboring cells and starts signalling through PEPR1/2 receptors to activate defence-related genes (Figure 2b) [34^••^].

Ablation of cells outside the root meristem (in the elongation zone) also triggers a Ca^2+^ wave and an increase in ROS accumulation in cells close to the wound site. Similar to previous studies [13], this Ca^2+^ wave and its propagation partly depend on enzymatic ROS production in the apoplast [37^••^]. These phenomena also coincide with a membrane depolarization close to the ablation site which probably comes from changed ion fluxes, like Ca^2+^and other available ions (Figure 2a) [37^••^].

Ablation experiments in the shoot apical meristem induce similar Ca^2+^ waves, which are required for the repolarization of the auxin efflux transporter PIN1 away from the wounded tissues [38], consistent with previously established importance of Ca^2+^ signalling for PIN polarity in roots [39]. Additionally, microtubules rearrange in the same cells after ablation as a consequence of a changed mechanical stresses, but this seems to be independent of the Ca^2+^ waves, indicating more complex and yet unknown mechanosensitive signalling mechanisms responsive to wounding [38].

### Involvement of phytohormones

As expected, multiple phytohormones are involved in coordinating regenerative processes following wounding but their exact role and interactions are far from clarified. Cell ablation or infection with root-invading nematodes, which can lead to the specific removal of single cells in the root, leads to the increase of the transcriptional ethylene response marker ACS6 as early as three hours after ablation. Defence against these invaders depends on ethylene signalling through EIN2 [37^••^,^40^] and this triggering of the ethylene signalling partly depends on the Ca^2+^ wave and ROS production by apoplast-localized oxidases. Overall, these observations reveal an important role of ethylene in the root immune and wound response (Figure 2a) [37^••^].

Jasmonates (JA), phytohormones typically associated with plant immunity, are induced around wounds specifically in the central root meristem as early as 30 s after the ablation (Figure 2d). Similarly, nematode infestation or root growing through rough soil inducs JA [41^••^]. Pending evidence to the contrary, it seems JA response is not induced in root tissues other than the root meristem [37^••^].

Auxin has been implicated among many other processes, also in regulation of division plane orientation, cell fate (re)specification [42] and for the maintenance of the stem cell niche in the root meristem centre [43]. Removal of the root tip triggers a strong auxin accumulation above the ablated cells, presumably due to a disruption of the intercellular auxin flow, to induce replacement of the meristem centre [44]. Chilling stress induces natural death in root tip cells, which thereby block auxin transport anatomically. The resulting auxin accumulation helps maintaining the meristem centre during the stress [45]. Increased auxin biosynthesis, in contrast, occurs in wounded leaves [25] and root stumps after meristem removal [46] and is crucial for the efficient tissue re-establishment. However, it remains unknown how wound-responsive auxin transport, biosynthesis or signalling play a role in local regenerative processes.

### Downstream transcriptional regulations

Besides the above-mentioned glimpses into wound-triggered signalling processes, little is known about the downstream mechanisms leading to regeneration. One of the few identified components is the ETHYLENE RESPONSE FACTOR 115 (ERF115), a transcription factor required for the efficient initiation of restorative divisions [31^••^,32^••^], and its close homologue and upstream regulator ERF109 [41^••^]. Without wounding, the ERF115 expression domain is usually restricted to the rarely occurring cell divisions in the quiescent centre, where it is controlled by brassinosteroids [47,48], but it can be slightly increased by exogenous application of ROS, auxin, and JA [41^••^,^49^]. In some cell types after wounding, ERF115 becomes upregulated in a JA-dependent manner during restorative divisions in cells directly adjacent to the wound (Figure 2d) [41^••^,31^••^]. It remains a mystery how such spatially restricted ERF115 induction is achieved by rather broadly spreading signals, exactly which factors are involved in cell types where ERF115 is not induced and which downstream targets of ERF115 mediate the regeneration.

## Conclusions

Several recent studies using the single cell ablation allowed identifying wound response processes at different levels: (i.) local – cells directly adjacent to the wounds, (ii.) regional – cell groups in close proximity, or (iii.) systemic – the whole tissue in the same organ or in completely different parts of the plants. Comparable responses after cell ablation, nematode infestation or naturally occurring wounding suggest that laser-assisted cell elimination can be used to study mechanism of wound healing.

Multiple signals have been identified to be involved in the response to wounding, but the nature of the primary wound signal which activates the adjacent cells remains completely elusive, along with most of the downstream regeneration mechanisms. Further studies, building up on these initial findings and combining laser-assisted cell elimination with live imaging, forward genetic screens and single cell transcriptomics will allow us to get detailed molecular insights of what is happening at the local, regional, and systemic levels and how different signalling mechanisms cooperatively contribute toward wound healing. These studies not only will reveal mechanisms of tissue regeneration but also help us to understand the general mechanisms of positional information-based tissue patterning.

## Conflict of interest statement

Nothing declared.

## References and recommended reading

Papers of particular interest, published within the period of review, have been highlighted as

• of special interest
•• of outstanding interest
